# Genetic Polymorphisms and Platinum-Based Chemotherapy-Induced Toxicities in Patients With Lung Cancer: A Systematic Review and Meta-Analysis

**DOI:** 10.3389/fonc.2019.01573

**Published:** 2020-03-17

**Authors:** Wenhui Liu, Ying Wang, Jianquan Luo, Haiyan Yuan, Zhiying Luo

**Affiliations:** ^1^Department of Pharmacy, The Second Xiangya Hospital, Central South University, Changsha, China; ^2^Institute of Clinical Pharmacy, Central South University, Changsha, China

**Keywords:** platinum, pharmacogenomics, toxicity, individual difference, meta-analysis

## Abstract

**Background:** Platinum-based agents, including cisplatin, carboplatin, and oxaliplatin, are indispensable for the treatment of lung cancer. The development of toxicity frequently necessitates dose reduction or discontinuation of therapy, despite the clinical response. Pharmacogenomics studies were reviewed to identify the possible genetic variants that underlie individual susceptibility to platinum-related toxicities.

**Method:** We conducted a systematic search in PubMed and Embase for pharmacogenomics reports that focused on commonly reported platinum-induced toxicities, such as gastrointestinal (GI), hematological, neurological, and other toxicities, in patients diagnosed with lung cancer. Meta-analyses were conducted to determine the association between genetic polymorphisms and platinum-induced toxicity by checking the odds ratio (OR) and 95% confidence interval (CI) using random or fixed-effects models as appropriate.

**Results:** Twenty eligible studies that met the inclusion criteria with sufficient data were extracted and presented comprehensively. A total of 16 polymorphisms from 11 genes were included in the meta-analysis. *MTHFR* rs1801131 and *MDM2* rs1690924 were significantly correlated with platinum-induced GI toxicity (*P* = 0.04 and *P* = 0.02, respectively). Patients with the *MTHFR* rs1801131AA and *MDM2* rs1690924TC/CC genotype tended to have a higher risk of GI toxicity than patients with other genotypes did (OR = 1.73, 95% CI = 0.86–2.18; and OR = 0.51, 95% CI = 0.29–0.88, respectively). Compared to carriers of the *MTHFR* rs1801133CC genotype, carriers of the CT/TT genotype had a significantly increased risk of hematological toxicity (*P* = 0.01, OR = 1.68, 95% CI = 1.12–2.52).

**Conclusion:** In the future, physicians should pay careful attention to *MTHFR* and *MDM2* for personalized chemotherapy treatment among patients with lung cancer.

## Background

Lung cancer is the second most commonly diagnosed malignant tumor in men and women and is one of the main causes of mortality worldwide (1). There are two major forms of lung cancer, including small cell lung cancer (SCLC) and non-small cell lung cancer (NSCLC); NSCLC accounts for 85% of all cases of lung cancer (2). Because of the difficulty in early diagnosis, most patients are diagnosed with advanced stage disease when surgery is no longer a treatment option. Therefore, chemotherapy is the major treatment choice for these patients (3).

Platinum-based agents, including cisplatin, carboplatin, and oxaliplatin, in combination with other cytotoxic drugs have been recommended as the first-line chemotherapy for lung cancer (4). The antitumor effect of platinum-based agents is by interfering with DNA repair, thereby suppressing and eventually killing cancer cells (5). Unfortunately, platinum can also hamper the growth of normal cells. Although platinum-based agents are effective for cancer therapy, platinum-induced toxicity is very common in clinical settings. The use of platinum-based agents may lead to serious or permanent adverse events, such as hematotoxicity, GI, nephrotoxicity, hearing loss, and other toxicities (6). Severe toxicities can result in dose reduction, treatment delay, or even chemotherapy discontinuation, as well as carry the risk of life-threatening complications (7). Moreover, differences exist among patients considering the severity of platinum-induced toxicities. Therefore, personalized medicine aims to identify patients who have received platinum agent treatment and are more likely to benefit from anticancer agents or more likely to experience adverse events.

Genetic polymorphisms contribute to the differences in platinum-related toxicities, and there is accumulating evidence to support this speculation (8). Therefore, determining the association between polymorphisms and platinum-related toxicities will be beneficial for individualized chemotherapy. Previously, two genome-wide association studies and one whole-exome sequencing study were conducted to identify the genetic markers for platinum-induced toxicities (9–11). In addition, Yin et al. aimed to establish models to explain and predict platinum toxicity interindividual difference by simultaneously incorporating multiple genetic and clinical factors to explore the association of their interactions with platinum-induced toxicities (12, 13).

Although many studies have investigated this issue, there is still no consensus regarding the relationship between genetic polymorphisms and platinum-induced toxicities in patients with lung cancer. For example, Cristina et al. found that *ERCC1* C118T was significantly associated with platinum-induced toxicity while other studies presented contradictory results (14–16). Similar associations were found for *XRCC1* codon 399, *XPD* Lys751Gln, and other mutations (17, 18). Hence, quantitative evaluation is needed for determining the association between gene polymorphisms and platinum-induced toxicities.

The aims of this study were as follows: (1) to summarize the pharmacogenomics of platinum-based toxicities in patients with lung cancer and (2) to provide a comprehensive assessment of the association between genetic polymorphisms and platinum-based drug response in patients with lung cancer. We collected all available publications on pharmacogenomics studies that platinum-based toxicities in patients with NSCLC and SCLC and quantitatively studied them using a meta-analysis strategy.

## Methods

### Search Strategy

For the literature search, two authors (Z. Y. Luo and W. H. Liu) independently performed a systematic literature search in three databases: PubMed database, Cochrane Library, and ISI Web of Knowledge. The search results were reviewed and compared by a third reviewer (Y. Zheng), and discrepancies between searchers were discussed and solved with consensus. The literature was searched from the first available article to June 18, 2019. Publications were retrieved using terms associated with platinum drugs (“platinum” or “cisplatin” or “carboplatin” or “oxaliplatin”) in combination with keywords associated with genetic variation (“polymorphism” or “SNP” or “single nucleotide polymorphism” or “mutation” or “variation”) and “toxicity” or “adverse effect,” and “lung cancer.”

### Inclusion Criteria

All identified abstracts were carefully and independently reviewed by two investigators (Z. Y. Luo and W. H. Liu) for eligibility. The inclusion criteria were as follows: (i) clinical studies, regardless of the sample size; (ii) studies that assessed the associations between genetic polymorphisms and platinum-induced severe (grade 3–4) toxicities in patients with lung cancer, and toxicities were evaluated and graded according to the Common Terminology Criteria for Adverse Events; (iii) numbers for each genotype were available or could be calculated in different groups; and (iv) at least two studies that evaluated one polymorphism met the abovementioned three criteria. If the two investigators disagreed about the eligibility of an article, it was resolved by consensus with a third reviewer (J. Q. Luo).

### Data Extraction

Data were manually extracted by two reviewers (Z. Y. Luo, W. H. Liu) who were blinded to each other and used the same data recording form. All data were reviewed by the third reviewer (J. Q. Luo) until they reached a consensus on all of the data extraction items. The following information was extracted from each study: name of the first author, publication year, country of the study and ethnicity of the patients, sample size, tumor type, disease stage, chemotherapeutic drugs, platinum-induced toxicities, toxicity evaluation criteria, genes and Single Nucleotide Polymorphism Database number of the investigated polymorphisms, and genotype methods.

### Statistical Analysis

The patients were divided into two groups: those with grade 3–4 (severe) toxicities and those with grade 0–2 (no or mild) toxicities. The pooled odds ratio (OR) and associated 95% CI were calculated and used to evaluate the strength of association in different genotype groups. The significance of the pooled estimates of the OR was determined using the *Z*-test. The Cochran's *Q*-test and *I*^2^ metric were performed to determine the possibility of between-study heterogeneity. The heterogeneity of publications in each meta-analysis was considered to be significant at *P* < 0.05 for the *Q* statistics and *I*^2^ > 50% for the *I*^2^ metric. Sensitivity analysis was conducted if publication bias existed; one study was excluded at a time, and the others were analyzed to estimate whether the results were affected markedly by a single study. Subgroup analysis based on toxicity [categorized as overall, gastrointestinal (GI), and hematological toxicities] was conducted to assess the sources of heterogeneity across the studies. Potential publication bias was assessed using the Begg and Egger tests, with *P* < 0.05 considered to indicate a significant publication bias. All statistical analyses were performed with by the Cochrane Collaboration software (Review Manager 5, the Cochrane Collaboration, Oxford, United Kingdom).

## Results

### Literature Review and Characteristics of the Included Studies

The initial research yielded 1,003 publications. A total of 608 articles were excluded owing to duplicate publication; in addition, 306 studies were excluded in the first round of review, among which 30 were irrelevant literature articles, 5 were not English articles, 64 were reviews or meta-analysis, 27 were case reports or abstracts, 19 were non-clinical-related studies, 13 focused on other tumors, 41 did not evaluate platinum-based chemotherapy, 20 did not focus on platinum-related toxicities, and 89 were not pharmacogenomic studies. After reading the full text of these articles, we found that 12 studies did not provide detailed data owing to the lack of significant association between gene polymorphisms and platinum-based toxicities.

**Figure 1:**
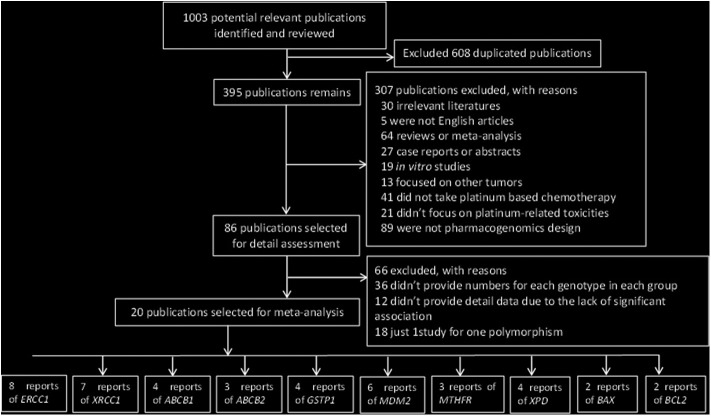
Flow chart of literature selection.

Finally, 20 publications with sufficient data met the inclusion criteria and were extracted (Figure 1). The general characteristics of the studies included in the meta-analysis are presented in Table 1. The included publications included 16 polymorphisms in 11 genes, and the detailed information of these mutation are listed in Table 2. A candidate gene approach was employed for the identification of single nucleotide polymorphisms (SNPs) that conferred susceptibility to platinum-based toxicities, and this methodology directly evaluated the relationship between one variant and a particular toxicity or several toxicities.

**Table 1:** Characteristics of studies involved in the meta-analysis.

**Authors**	**Year**	**Country**	***N***	**Disease stage**	**Cancer type**	**Toxicity evaluation**	**Genotype method**	**Genes and SNP**	**References**
Beatrice et al.	2019	Italy	82	II–IV	NSCLC + SCLC	CTCAE v4.03	TaqMan	*ABCB1*: rs1045642 *ABCB2*: rs717620 *GSTP1*: rs1695	(19)
Wang et al.	2018	China	490	III–IV	NSCLC	NCI-CTC 3.0	MALDI-TOF mass spectrometer	504 SNPs of 185 genes	(12)
Zheng et al.	2017	China	1218	IIIa–IV	NSCLC	NCI-CTC 3.0	MassARRAY	*ERCC1*: rs11615, rs3212986 *XRCC1*: rs25487 *MDM2*: rs2279744	(20)
Qian et al.	2016	China	403	I–IV	NSCLC	NCI-CTC 3.0	MassARRAY	*ABCB1*: rs1045642, *ABCB2*: rs717620	(21)
Cristina et al.	2016	Spain	141	I–IV	NSCLC	NCI-CTCAE 4.0	TaqMan	*ERCC1*: rs11615, rs3212986 *XRCC1*: rs25487 *ABCB1*: rs1045642 *MDM2*: rs1470383, rs1690924 *MTHFR*: rs1801131, rs1801133	(14)
Powrozek et al.	2016	Poland	55	IIIb–IV	NSCLC	NCI-CTC 4.03	Mini-sequencing	*ERCC1*: rs11615, rs3212986 *XRCC1:* rs25487	(18)
Deng et al.	2015	China	97	IIIb–IV	NSCLC	CTCAE, V2.0	Pyrosequencing	*XRCC1*: rs25487	(22)
Qian et al.	2015	China	663	IIIa–IV	NSCLC	NCI-CTC 3.0	iSelect HD BeadChip	*MDM2*: rs1470383, rs1690924	(23)
Peng et al.	2015	China	235	III–IV	NSCLC	NCI-CTC 3.0	PCR-RFLP	BAX: rs4645878 BCL2: rs2279115	(24)
Li et al.	2014	China	1004	III–IV	NSCLC	NCI-CTC 3.0	iSelect HD BeadChip	*MTHFR*: rs1801133, rs1801131	(25)
Peng et al.	2014	China	235	III–IV	NSCLC	NCI-CTC 3.0	PCR-CTTP	*XRCC1*: rs25487	(26)
Wang et al.	2014	China	119	NA	SCLC	NCI-CTCAE 3.0	MassARRAY	*MDM2*: rs2279744	(27)
Zheng et al.	2014	China	444	IIIa–IV	NSCLC	NCI-CTC 3.0	PCR-RFLP	*MDM2:* rs2279744, *MDM2*: rs937282	(28)
Gu et al.	2012	China	445	IIIa–IV	NSCLC	NCI-CTC 3.0	MALDI-TOF mass spectrometer	BAX: rs4645878 BCL2: rs2279115	(29)
Markus et al.	2011	Switzerland	137	IIIb–IV	NSCLC	NCI-CTC 3.0	sequencing	*GSTP1*: rs1695	(15)
Vienna et al.	2011	Italy	192	IIIb–IV	NSCLC	NCI-CTC 3.0	TaqMan	*XPD*: rs13181	(30)
Wang et al.	2008	China	139	IIIb–IV	Advanced lung cancer	NCI-CTC 3.0	PCR-RFLP	*XRCC1*: rs25487	(31)
Carmelo et al.	2008	Italy	65	IIIb–IV	NSCLC	NCI-CTC 3.0	Taqman	*ERCC1*: rs11615 *XPD*: rs13181, rs1799793	
Richard et al.	2006	United Kingdom	108	III–IV	NSCLC	NCI-CTC 2.0	Sequencing	*GSTP1*: rs1695	(32)
Rebecca et al.	2005	USA	214	III–IV	NSCLC	NCI-CTC 3.0	Taqman	*ERCC1*: rs11615, rs3212986	(33)

*NCI-CTC, National Cancer Institute Common Toxicity Criteria; NCI-CTCAE, National Cancer Institute Common Terminology Criteria for Adverse Events; PCR-CTTP, PCR with confronting two-pair primers; PCR-RFLP, PCR-based restriction fragment length polymorphism*.

**Table 2:** Polymorphisms and phenotypes analyzed in this study.

**Genes**	**Polymorphisms**	**NCBI ID**	**Alleles**	**Platinum-related toxicities**	**References**
ERCC1	C118T (Asn118Asn)	rs11615	C>T	Grade 3–4 hematological toxicity	(12, 14, 20, 30, 33, 34)
				Grade 3–4 GI toxicity	(12, 14, 33)
	C8092A	rs3212986	C>A	Grade 3–4 hematological toxicity	(12, 14, 18, 20)
				Grade 3–4 nephrotoxicity toxicity	(14, 18)
XRCC1	G1196A (Arg399Gln)	rs25487	G>A	Grade 3–4 GI toxicity	(12, 14, 20, 26, 31)
				Grade 3–4 hematological toxicity	(12, 14, 20, 26, 31)
P53	Arg72Pro	rs1042522	G>C	Grade 3–4 hematological toxicity	(12, 27, 28, 30)
ABCB1	G2677T/A (Ala893Ser/Thr)	rs1045642	G>T/A	Grade 3–4 overall toxicity	(12, 14, 21)
				Grade 3–4 hematological toxicity	(12, 14, 19, 21)
				Grade 3–4 GI toxicity	(12, 14, 21)
ABCB2	−24C>T	rs717620	C>T	Grade 3–4 hematological toxicity	(12, 19, 21)
GSTP1	A313G (Ile105Val)	rs1695	A>G	Grade 3–4 hematological toxicity	(12, 15, 19, 32)
XPD	A2251C (Lys751Gln)	rs13181	C>A	Grade 3–4 hematological toxicity	(12, 18, 30, 34)
	G934A (Asp312Asn)	rs1799793	G>A	Grade 3–4 hematological toxicity	(12, 18, 34)
MTHFR	A1298C	rs1801131	A>C	Grade 3–4 GI toxicity	(14, 25)
				Grade 3–4 hematological toxicity	(14, 25)
	C677T	rs1801133	C>T	Grade 3–4 GI toxicity	(12, 14, 25)
				Grade 3–4 hematological toxicity	(12, 14, 25)
MDM2	Intron variant	rs1470383	C>T	Grade 3–4 overall toxicity	(14, 23)
				Grade 3–4 hematological toxicity	(14, 23)
				Grade 3–4 GI toxicity	(14, 23)
	309T>G	rs2279744	G>T	Grade 3–4 hematological toxicity	(12, 20, 27, 28)
	Intron variant	rs1690924	C>T	Grade 3–4 overall toxicity	(14, 23)
				Grade 3–4 hematological toxicity	(14, 23)
				Grade 3–4 GI toxicity	(14, 23)
BAX	−248G>A	rs4645878	G>A	Grade 3–4 hematological toxicity	(24, 29)
				Grade 3–4 GI toxicity	(24, 29)
BCL2	938C>A	rs2279115	G>A	Grade 3–4 hematological toxicity	(24, 29)
				Grade 3–4 GI toxicity	(24, 29)

### Quantitative Synthesis of the Association Between Polymorphisms and Platinum-Related Toxicities

The main results of this meta-analysis showing the association between polymorphisms and risk of platinum-based grade 3–4 toxicities are shown in Table 3 and Supplemental Figures.

**Table 3:** Summary of meta-analysis of the association of genetic polymorphisms with platinum induced toxicities.

**Gene**	**SNP**	**Toxicity**	**Polled OR (95% CI)**	***Z***	***P***	***N***	**Model**	***I*^**2**^ (%)**	***P*_**hetero**_**
*ERCC1*	C118T	Grade 3–4 GI toxicity	0.77 [0.43, 1.38]	0.88	0.38	790	F	0	0.94
		Grade 3–4 hematological toxicity	0.80 [0.56, 1.15]	1.20	0.23	1,450	F	0	0.81
	C8092A	Grade 3–4 hematological toxicity	0.86 [0.65, 1.15]	1.01	0.31	1,037	F	0	0.88
		Grade 3–4 nephrotoxicity toxicity	0.88 [0.62, 1.25]	0.73	0.47	196	F	0	0.37
*XRCC1*	rs25487	Grade 3–4 hematological toxicity	0.94 [0.65, 1.35]	0.35	0.72	1,366	F	15	0.32
		Grade 3–4 GI toxicity	1.29 [0.53, 3.16]	0.56	0.57	1,366	R	71	0.009
*P53*	rs1042522	Grade 3–4 hematological toxicity	0.82 [0.59, 1.15]	1.15	0.25	1,033	F	30	0.23
*ABCB1*	rs1045642	Grade 3–4 overall toxicity	1.77 [0.79, 3.95]	1.39	0.16	1,045	R	87	0.0006
		Grade 3–4 hematological toxicity	1.97 [0.87, 4.47]	1.63	0.10	1,153	R	85	0.0002
		Grade 3–4 GI toxicity	1.34 [0.38, 4.75]	0.46	0.65	957	R	83	0.003
*ABCB2*	rs717620	Grade 3–4 hematological toxicity	1.35 [0.43, 4.25]	0.51	0.61	923	R	90	<0.0001
*GSTP1*	A313G	Grade 3–4 hematological toxicity	1.44 [0.77, 2.7]	1.14	0.26	745	F	0	0.49
*XPD*	rs13181	Grade 3–4 hematological toxicity	1.00 [0.55, 1.85]	0.01	0.99	742	F	0	0.75
	rs1799793	Grade 3–4 hematological toxicity	2.46 [0.46, 13.04]	1.06	0.29	548	R	72	0.03
*MTHFR*	rs1801131	Grade 3–4 GI toxicity	1.73 [0.86, 2.18]	2.02	0.04	1,106	F	24	0.25
		Grade 3–4 hematological toxicity	0.74 [0.44, 1.24]	1.14	0.26	1,231	F	0	0.94
	rs1801133	Grade 3–4 GI toxicity	1.29 [0.86, 1.92]	1.23	0.22	1,227	F	0	0.52
		Grade 3–4 hematological toxicity	1.68 [1.12, 2.52]	2.49	0.01	1,229	F	0	0.40
*MDM2*	rs2279744	Grade 3–4 hematological toxicity	0.69 [0.29, 1.62]	0.85	0.39	1,189	R	80	0.002
	rs1470383	Grade 3–4 overall toxicity	0.99 [0.71, 1.37]	0.07	0.95	786	F	0	0.48
		Grade 3–4 hematological toxicity	0.91 [0.64, 1.28]	0.56	0.57	786	F	35	0.21
		Grade 3–4 GI toxicity	1.35 [0.74, 2.46]	0.98	0.33	786	F	0	0.57
	rs1690924	Grade 3–4 overall toxicity	0.83 [0.60, 1.13]	1.19	0.23	786	F	0	0.74
		Grade 3–4 hematological toxicity	0.98 [0.70, 1.38]	0.10	0.92	755	F	7	0.30
		Grade 3–4 GI toxicity	0.51 [0.29, 0.88]	2.40	0.02	782	F	0	0.74
BAX	rs4645878	Grade 3–4 hematological toxicity	1.46 [0.96, 2.20]	1.79	0.07	647	F	0	0.52
		Grade 3–4 GI toxicity	1.15 [0.63, 2.09]	0.46	0.64	635	F	43	0.19
BCL2	rs2279115	Grade 3–4 hematological toxicity	1.00 [0.72, 1.39]	0.02	0.98	643	F	0	0.91
		Grade 3–4 GI toxicity	0.81 [0.48, 1.38]	0.78	0,44	632	F	0	0.62

*F, fixed-effects model; R, random model; P_hetero_, P-value for heterogeneity test*.

### *ERCC1* C118T and C8092A

The most extensively studied polymorphism was *ERCC1* C118T. The association between C118T polymorphism and platinum-based grade 3–4 hematological and GI toxicity was found and replicated by six and three studies, respectively. We first performed a meta-analysis to determine the association between C118T polymorphism and grade 3–4 hematological toxicity by including 1,450 subjects. No significant relationship was detected between C118T polymorphism and grade 3–4 hematological toxicity (OR = 0.80, 95% CI: 0.56–1.15, *P* = 0.23) using a fixed-effect model. On subgroup analyses based on ethnicity, the combined OR for risk and the *I*^2^ were consistent and did not show any apparent fluctuation (Supplemental Table 1).

We further analyzed the relationship between C118T polymorphism and grade 3–4 GI toxicity by including 790 patients. Pooled data from these investigations showed grade 3–4 GI toxicity rates of 13.49 and 14.15% in the CC + CT genotype and the TT genotype groups, respectively. No significant relationship was detected between C118T polymorphism and grade 3–4 GI toxicity (OR = 0.77, 95% CI: 0.43–1.38, *P* = 0.38) using a fixed-effect model. This association did not significantly change on subgroup analyses based on ethnicity.

A total of four and two studies examined the association between C8092A mutation and platinum-based grade 3–4 hematological toxicity and nephrotoxicity, respectively. No significant association was detected between C8092A polymorphism and grade 3–4 hematological toxicity (OR = 0.86, 95% CI: 0.65–1.15, *P* = 0.31) using a fixed-effect model. This association had no significant change on subgroup analyses based on ethnicity. Among 196 patients who were included to determine the association between C8092A polymorphism and grade 3–4 nephrotoxicity, no significant association was observed (OR = 0.88, 95% CI: 0.62–1.25, *P* = 0.58) using a fixed-effect model.

### *XRCC1* G1196A

Five studies were eligible to determine the association between G1196A polymorphism and grade 3–4 GI and hematological toxicities. Pooled analysis showed that there was no significant association between G1196A polymorphism and grade 3–4 GI toxicity (OR = 1.29, 95% CI: 0.53–3.16, *P* = 0.56) using a random-effect model (Table 3 and Supplemental Figure 2). A sensitivity analysis was conducted because of publication bias (*I*^2^ = 71%). After removing the study by Peng et al., the publication bias disappeared (*I*^2^ = 10%). We further examined the raw data of this study and found that the G1196A mutation was not considered in the Hardy–Weinberg equation (*P* = 0.002). No significant association was detected between G1196A polymorphism and grade 3–4 hematological toxicity (OR = 0.94, 95% CI: 0.65–1.35, *P* = 0.35) using a fixed-effect model. On subgroup analyses based on ethnicity, the combined risk of G1196A mutation on platinum-based grade 3–4 GI toxicities was consistent and did not show any apparent fluctuation (Supplemental Table 1).

### *P53* Arg72Pro

A total of four studies with 1,033 patients were included to determine the association between *P53* Arg72Pro polymorphism and grade 3–4 hematological toxicity. However, the pooled analysis showed no significant association between Arg72Pro polymorphism and grade 3–4 hematological toxicity (OR = 0.82, 95% CI: 0.59–1.15, *P* = 0.25) using a fixed-effect model. This association did not significantly change on subgroup analyses based on ethnicity (Supplemental Table 1).

### *ABCB1* G2677T/A

A total of three, four, and three studies were available to determine the association between G2677T/A polymorphism and platinum-induced grade 3–4 overall, hematological, and GI toxicities, respectively. On overall analysis, no significant association was detected between G2677T/A polymorphism and grade 3–4 overall toxicity (OR = 1.77, 95% CI: 0.79–3.95, *P* = 0.16) using a random-effect model. In addition, publication bias disappeared (*I*^2^ = 0%) after the study by Wang et al. was removed. The possible reason is that the call rate for G2677T/A genotypes was <90% in their study, which could have led to inaccuracy in their results. On subgroup analyses based on ethnicity, different ethnic populations showed distinct effects for the G2677T/A polymorphism. There were significant protective effects of the T/A allele on the risk of platinum-based overall toxicity in the non-Chinese subgroup (Supplemental Table 1).

Moreover, no significant association was detected between the G2677T/A polymorphism and grade 3–4 hematological toxicity (OR = 1.97, 95% CI: 0.87–4.47, *P* = 0.10) using a random-effect model. After removing the study by Qian et al., publication bias disappeared (*I*^2^ = 17%). Furthermore, the association between the G2677T/A polymorphism and grade 3–4 GI toxicity was analyzed. No significant association was detected between the G2677T/A polymorphism and grade 3–4 GI toxicity (OR = 1.34, 95% CI: 0.38–4.75, *P* = 0.46) using a random-effect model. In addition, the publication bias vanished (*I*^2^ = 0%) when the study by Qian et al. was removed. The study by Qian et al. was a high-quality well-designed study, with the appropriate sample size. The association between the G2677T/A polymorphism and grade 3–4 hematological and GI toxicities was still non-significant after the study by Qian et al. was removed.

### *ABCB2* −24C>T

Only three studies were qualified for analyzing the association between the −24C>T polymorphism and platinum-induced grade 3–4 hematological toxicity. No significant association was observed between the −24C>T polymorphism and grade 3–4 hematological toxicity (OR = 1.35, 95% CI: 0.43–4.25, *P* = 0.51) using a random-effect model. In addition, the publication bias vanished (*I*^2^ = 0%) when the study by Wang et al. was removed. The call rate for the −24C>T genotype was 83% in this study, which could have led to inaccuracy in the result. On subgroup analyses based on ethnicity, the association between the −24C>T polymorphism and grade 3–4 hematological toxicity was still non-significant (Supplemental Table 1).

### *GSTP1* A313G

Data from 745 patients included in 4 studies were used for analyzing the association between the A313G polymorphism and platinum-induced grade 3–4 hematological toxicity. There was no significant relationship between the A313G polymorphism and grade 3–4 hematological toxicity (OR = 1.44, 95% CI: 0.77–2.70, *P* = 0.26) using a fixed-effect model. On subgroup analyses based on ethnicity, the association between the A313G polymorphism and grade 3–4 hematological toxicity was still non-significant (Supplemental Table 1).

### *XPD* A2251C and G934A

A total of four studies determined the association between the A2251C polymorphism and grade 3–4 hematological toxicity. No significant correlation was detected between the A2251C polymorphism and grade 3–4 hematological toxicity (OR = 1.00, 95% CI: 0.55–1.85, *P* = 0.99) using a fixed-effect model. Three studies examined the association between the G934A polymorphism and grade 3–4 hematological toxicity. No significant association was detected between the G934A polymorphism and grade 3–4 hematological toxicity (OR = 2.46, 95% CI: 0.46–13.04, *P* = 0.29) using a random-effect model. The publication bias disappeared (*I*^2^ = 0%) when the study by Tibaldi et al. or that by Powrozek et al. was removed. The main reason for this phenomenon was the small sample size of these two studies.

On subgroup analyses based on ethnicity, a non-significant association was found between these two polymorphisms and grade 3–4 hematological toxicity (Supplemental Table 1).

### *MTHFR* A1298C and C677T

In total, three included studies evaluated the association between the A1298C polymorphism and grade 3–4 GI and hematological toxicities. Carriers of the AA genotype had more severe GI toxicity than carriers of the AC + CC genotype did (OR = 1.73, 95% CI: 0.86–2.18, *P* = 0.04) on a fixed-effect model. However, no significant association was found between the A1298C polymorphism and grade 3–4 hematological toxicities (OR = 0.74, 95% CI: 0.44–1.24, *P* = 0.26) using a fixed-effect model.

Two studies evaluated the association between the C677T polymorphism and grade 3–4 GI and hematological toxicities. No significant association was detected between the C677T polymorphism and grade 3–4 GI toxicity (OR = 1.29, 95% CI: 0.86–1.92, *P* = 0.22) using a fixed-effect model. The pooled data showed that patients with the 677CC genotype had an increased risk of severe hematological toxicity than the carriers of the CT + TT genotype did (OR = 1.86, 95% CI: 1.12–2.52, *P* = 0.01) using a fixed-effect model. The association between these two polymorphisms and grade 3–4 hematological toxicity was unchanged on subgroup analyses based on ethnicity (Supplemental Table 1).

### *MDM2* rs1470383, rs2279744, and rs1690924

Four studies with 1,189 Chinese patients evaluated the correlation between the rs2279744 polymorphism and grade 3–4 hematologic toxicity. There was no significant correlation between the rs2279744 polymorphism and grade 3–4 hematological toxicity (OR = 0.69, 95% CI: 0.29–1.62, *P* = 0.39) using a random-effect model. Similarly, we investigated the influence of a single study on the overall risk by excluding one study at a time. However, the combined overall risk and *I*^2^ were consistent and did not show any apparent fluctuation.

Data from 786 subjects in 2 studies were used for analyzing the association between the rs1470383 polymorphism and platinum-based toxicities, including grade 3–4 overall, hematological, and GI toxicities. The pooled results showed no significant correlations between the rs1470383 polymorphism and grade 3–4 overall, hematological, and GI toxicities.

Two studies were used for analyzing the association between the rs1690924 polymorphism and platinum-based toxicities, including grade 3–4 overall, hematological, and GI toxicities. The pooled results showed no significant correlations between the rs1470383 polymorphism and grade 3–4 overall and hematological toxicities. The pooled results from all patients indicated that the carriers of the TT + TC genotype had a markedly increased risk of grade 3–4 GI toxicity than carriers of the CC genotype did (OR = 0.51, 95% CI: 0.29–2.43, *P* = 0.02) using a fixed-effect model.

### *BAX* rs4645878

Two studies were included to analyze the association between the rs4645878 polymorphism and grade 3–4 hematological and GI toxicities. The pooled analysis showed no significant difference between the rs4645878 mutation and grade 3–4 hematological and GI toxicities using a fixed-effect model.

### *BCL2* rs2279115

The two studies that were included to the determine association between the rs4645878 polymorphism and grade 3–4 hematological and GI toxicities were included to evaluate the association between the rs2279115 polymorphism and grade 3–4 hematological and GI toxicities. The pooled analysis showed no significant differences between the rs4645878 mutation and grade 3–4 hematological and GI toxicities using a fixed-effect model.

## Discussion

Researchers had intense interest in the influence of genetic factors on the platinum-based adverse events considering interindividual differences. In the present meta-analysis, we included 20 studies with 6,287 patients with lung cancer who were treated with platinum-based regiments, and we systematically evaluated the impact of genetic polymorphisms on platinum-based grade 3–4 toxicities. A total of 16 polymorphisms in 11 genes were analyzed, and our results provided evidence that *MTHFR* A1298C and C677T and *MDM2* rs1470383 polymorphisms were significantly associated with platinum-based grade 3–4 toxicities.

To date, platinum-based chemotherapy is widely used as first-line therapy for the treatment of lung cancer and is highly cost-effective in Chinese patients (35). As the cytotoxic effects of platinum are not specific, the mechanism of toxicity appears to involve multiple systems during chemotherapy. In the current study, we found that A1298C and C677T mutations of *MTHFR* were significantly associated with platinum-based grade 3–4 GI and hematological toxicities, respectively. *MTHFR* encodes the methylenetetrahydrofolate reductase enzyme that is involved in the folate metabolism pathway. The folate metabolism pathway plays an essential role in platinum cytotoxicity, and *MTHFR* is essential for transmethylation reactions including DNA methylation and DNA synthesis, thereby contributing to cancer prognosis (36). Both A1298C and C677T polymorphisms were associated with reduced enzyme activity and correlated with DNA hypomethylation, both of which alter the sensitivity of tumor cell to platinum compounds (37).

The gene encoding murine double minute 2 (*MDM2*) is a proto-oncogene and a key negative regulator of p53. *MDM2* plays a role in P53-independent antitumor activity through directing binding, ubiquitination, and degradation of the *p53* gene (38). A previous meta-analysis found that the *MDM2* gene polymorphism (rs2279744) was associated with the risk of lung cancer and the clinical outcomes (39). A previous study verified that unnatural change in the expression of *MDM2*, mediated by polymorphisms, contributed to subsequent attenuation of the p53 pathway, thereby accelerating the spread of NSCLC (40). In the current study, the *MDM2* rs1690924 mutation was significantly associated with grade 3–4 GI toxicity, and the carriers of the TT + TC genotype had a markedly increased risk of grade 3–4 GI toxicity than the carriers of the CC genotype did. To date, only one study found an association between this polymorphism and overall survival (41). However, the function of *MDM2* rs1690924 polymorphism is unknown.

Findings on genetic polymorphisms that affect platinum-induced toxicities were inconclusive in most studies, and the sample size of most studies was generally small. Polymorphisms in genes encoding the nucleotide excision repair (NER) pathway are among the most clinically relevant genetic determinants with susceptibility to platinum-based toxicities (42). The NER pathway is responsible for repairing DNA intrastrand cross-links induced by platinum-based chemotherapy, and the most commonly reported candidate gene associated with platinum-based toxicities include *ERCC1, XRCC1*, and *XPD* (43). In fact, genes involved in the NER pathway were most commonly evaluated in this meta-analysis. However, results from our meta-analysis showed no significant association between the gene polymorphisms of the NER pathway and platinum-induced severe toxicities.

Although previous studies validated that polymorphisms in genes act as potential risk factors for platinum-induced toxicities considering individual differences, the results of our research indicated that some polymorphisms correlated with platinum-induced toxicities had limited contribution to the interindividual differences in platinum-induced toxicities. The reasons for negative or conflicting results may be complicated. (i) The incidence of toxicities between cisplatin, carboplatin, and oxaliplatin varied (44). (ii) Although all patients in these studies were receiving treatment with platinum-based drugs, the use of non-platinum drugs, such as antimicrotubule agents, antifolate agents, or pyrimidine antagonists, as part of the chemotherapy regimens can affect toxicities profiles (45). (iii) The evaluation of toxicities was based on a standard handbook, while bias may have occurred when certain toxicities such as GI toxicity mainly depend on the subjective assessment by physicians. (iv) Platinum-induced toxicities were aggravated by the cumulative dosage; therefore, different chemotherapy cycles may also affect the results. (v) Platinum-induced toxicities may be affected by other factors, such as the demographic characteristics, molecular features of tumors, comorbidity, ethnicity, and intestinal bacteria.

Till date, the candidate gene approach is the most widely used strategy to identify platinum-induced toxicities and their associated polymorphisms. Novel variants will not be identified using this method, although the likelihood of a positive or negative association with a particular adverse event may be considerable in robust studies. Nevertheless, although previous GWAS and whole-exon sequencing had found novel genetic factors associated with platinum-induced toxicities, all these identified interactions need further verification to determine the mechanism.

The current meta-analysis has several limitations. First, studies without detailed data were excluded because of the lack of information regarding a significant association between gene polymorphisms and platinum-induced toxicities, and we were unable to contact these authors to provide us with the detailed data. This may have caused publication bias. Second, some of the included studies had small sample sizes, which may result in a decreased power to detect significant differences in the distribution of genotypes between grade 3–4 toxicities and grade 0–2 toxicities. Third, the occurrence of platinum-induced toxicities is affected by various factors, and hence, the pooled OR in this meta-analysis was based on the crude OR from the original studies. Because we could not obtain the raw data from individual studies, the pooled OR in this study was not adjusted for potential confounding factors such as sex, age, smoking status, comedications, gene–gene interactions, and gene–environment interactions, among other factors.

The results of our study indicated that single grade 3–4 toxicities that were associated with genetic polymorphisms might partly be the cause for inter-individual differences. Hence, further pharmacogenomics research is needed to determine the novel mutations and their associations. The combined effect of the genetic and clinical factors via gene–gene and gene–environment interactions should be considered in future studies, which may help to predict the risk of lung cancer. Nevertheless, more number of prospective, high-quality, multicenter clinical trials are urgently needed to explore the impact of gene polymorphisms on platinum-based toxicities in patients with lung cancer.

## Conclusion

Our study found that *MTHFR* A1298C and C677T polymorphisms and *MDM2* rs1470383 polymorphisms were significantly correlated with platinum-induced severe toxicities in patients with lung cancer. These polymorphisms should be considered for personalized chemotherapy treatment for lung cancer in the future.

## Data Availability Statement

The raw data supporting the conclusions of this article will be made available by the authors, without undue reservation, to any qualified researcher.

## Author Contributions

ZL and WL conceived and designed the study. ZL and YW performed research. ZL and JL conducted data analysis. ZL, HY, and WL accomplishment the manuscript, reference collection, data management, statistical analyses, paper writing, and study design.

### Conflict of Interest

The authors declare that the research was conducted in the absence of any commercial or financial relationships that could be construed as a potential conflict of interest. The reviewer J-YY declared a shared affiliation, with no collaboration, with the authors to the handling editor at the time of review.

## References

[1] Siegel RL Miller KD Cancer statistics, 2019. J Clin. (2019) 69:7–34. 10.3322/caac.21551 30620402

[2] Herbst RS Heymach JV Lippman SM. Lung cancer. N Engl J Med. (2008) 359:1367–80. 10.1056/NEJMra0802714 18815398 PMC10662965

[3] Lebwohl D Canetta R. Clinical development of platinum complexes in cancer therapy: an historical perspective and an update. Eur J Cancer. (1998) 34:1522–34. 10.1016/s0959-8049(98)00224-x 9893623

[4] Novello S Barlesi F Califano R Cufer T Ekman S Levra MG. Metastatic non-small-cell lung cancer: ESMO Clinical Practice Guidelines for diagnosis, treatment and follow-up. Ann Oncol. (2016) 27:v1–27. 10.1093/annonc/mdw326 27664245

[5] Shi Y Sun Y. Medical management of lung cancer: experience in China. Thorac Cancer. (2015) 6:10–6. 10.1111/1759-7714.12168 26273329 PMC4448475

[6] Pilkington G Boland A Brown T Oyee J Bagust A Dickson R A systematic review of the clinical effectiveness of first-line chemotherapy for adult patients with locally advanced or metastatic non-small cell lung cancer. Thorax. (2015) 70:359–67. 10.1136/thoraxjnl-2014-205914 25661113

[7] Jiang N Chen XC Zhao Y. Analysis of the risk factors for myelosuppression after concurrent chemoradiotherapy for patients with advanced non-small cell lung cancer. Support Care Cancer. (2013) 21:785–91. 10.1007/s00520-012-1580-y 22936496

[8] Xiong Y Huang BY Yin JY. Pharmacogenomics of platinum-based chemotherapy in non-small cell lung cancer: focusing on DNA repair systems. Med Oncol. (2017) 34:48. 10.1007/s12032-017-0905-6 28215024

[9] Cao S Wang S Ma H Tang S Sun C Dai J. Genome-wide association study of myelosuppression in non-small-cell lung cancer patients with platinum-based chemotherapy. Pharmacogenomics J. (2016) 16:41–6. 10.1038/tpj.2015.22 25823687

[10] Green H Hasmats J Kupershmidt I Edsgard D de Petris L Lewensohn R. Using whole-exome sequencing to identify genetic markers for carboplatin and gemcitabine-induced toxicities. Clin Cancer Res. (2016) 22:366–73. 10.1158/1078-0432.CCR-15-0964 26378035

[11] Cao S Wang C Ma H Yin R Zhu M Shen W. Genome-wide association study on platinum-induced hepatotoxicity in non-small cell lung cancer patients. Sci Rep. (2015) 5:11556. 10.1038/srep11556 26100964 PMC4477405

[12] Wang LY Cui JJ Liu JY Guo AX Zhao ZY Liu YZ. Gene-gene and gene-environment interaction data for platinum-based chemotherapy in non-small cell lung cancer. Sci Data. (2018) 5:180284. 10.1038/sdata.2018.284 30531820 PMC6289114

[13] Cui JJ Wang LY Zhu T Gong WJ Zhou HH Liu ZQ. Gene-gene and gene-environment interactions influence platinum-based chemotherapy response and toxicity in non-small cell lung cancer patients. Sci Rep. (2017) 7:5082. 10.1038/s41598-017-05246-8 28698656 PMC5505954

[14] Perez-Ramirez C Canadas-Garre M Alnatsha A Villar E Delgado JR Faus-Dader MJ. Pharmacogenetic predictors of toxicity to platinum based chemotherapy in non-small cell lung cancer patients. Pharmacol Res. (2016) 111:877–84. 10.1016/j.phrs.2016.08.002 27498158

[15] Joerger M Burgers SA Baas P Smit EF Haitjema TJ Bard MP. Germline polymorphisms in patients with advanced nonsmall cell lung cancer receiving first-line platinum-gemcitabine chemotherapy: a prospective clinical study. Cancer. (2012) 118:2466–75. 10.1002/cncr.26562 22031394

[16] Gandara DR Kawaguchi T Crowley J Moon J Furuse K Kawahara M. Japanese-US common-arm analysis of paclitaxel plus carboplatin in advanced non-small-cell lung cancer: a model for assessing population-related pharmacogenomics. J Clin Oncol. (2009) 27:3540–6. 10.1200/jco.2008.20.8793 19470925 PMC2717760

[17] Li Y Huang XE Jin GF Shen HB Xu L Lack of any relationship between chemotherapy toxicity in non-small cell lung cancer cases and polymorphisms in XRCC1 codon 399 or XPD codon 751. Asian Pac J Cancer Prev. (2011) 12:739–42. 21627375

[18] Powrozek T Mlak R Krawczyk P Homa I Ciesielka M Koziol P. The relationship between polymorphisms of genes regulating DNA repair or cell division and the toxicity of platinum and vinorelbine chemotherapy in advanced NSCLC patients. Clin Transl Oncol. (2016) 18:125–31. 10.1007/s12094-015-1343-6 26193985

[19] De Troia B Dalu D Filipazzi V Isabella L Tosca N Ferrario S. ABCB1 c.3435C>T polymorphism is associated with platinum toxicity: a preliminary study. Cancer Chemother Pharmacol. (2019) 83:803–8. 10.1007/s00280-019-03794-6 30796464

[20] Zheng Y Deng Z Yin J Wang S Lu D Wen X. The association of genetic variations in DNA repair pathways with severe toxicities in NSCLC patients undergoing platinum-based chemotherapy. Int J Cancer. (2017) 141:2336–47. 10.1002/ijc.30921 28791697

[21] Qian CY Zheng Y Wang Y Chen J Liu JY Zhou HH. Associations of genetic polymorphisms of the transporters organic cation transporter 2 (OCT2), multidrug and toxin extrusion 1 (MATE1), and ATP-binding cassette subfamily C member 2 (ABCC2) with platinum-based chemotherapy response and toxicity in non-small cell lung cancer patients. Chin J Cancer. (2016) 35:85. 10.1186/s40880-016-0145-8 27590272 PMC5010769

[22] Deng JH Deng J Shi DH Ouyang XN Niu PG. Clinical outcome of cisplatin-based chemotherapy is associated with the polymorphisms of GSTP1 and XRCC1 in advanced non-small cell lung cancer patients. Clin Transl Oncol. (2015) 17:720–6. 10.1007/s12094-015-1299-6 26033426

[23] Qian J Liu H Gu S Wu Q Zhao X Wu W. Genetic variants of the MDM2 gene are predictive of treatment-related toxicities and overall survival in patients with advanced NSCLC. Clin Lung Cancer. (2015) 16:e37–53. 10.1016/j.cllc.2015.02.001 25818095

[24] Peng Y Wang L Qing Y Li C Ren T Li Q. Polymorphisms of BCL2 and BAX genes associate with outcomes in advanced non-small cell lung cancer patients treated with platinum-based chemotherapy. Sci Rep. (2015) 5:17766. 10.1038/srep17766 26656462 PMC4674711

[25] Li X Shao M Wang S Zhao X Chen H Qian J. Heterozygote advantage of methylenetetrahydrofolate reductase polymorphisms on clinical outcomes in advanced non-small cell lung cancer (NSCLC) patients treated with platinum-based chemotherapy. Tumour Biol. (2014) 35:11159–70. 10.1007/s13277-014-2427-6 25104092

[26] Peng Y Li Z Zhang S Xiong Y Cun Y Qian C. Association of DNA base excision repair genes (OGG1, APE1 and XRCC1) polymorphisms with outcome to platinum-based chemotherapy in advanced nonsmall-cell lung cancer patients. Int J Cancer. (2014) 135:2687–96. 10.1002/ijc.28892 24729390

[27] Wang X Wang YZ Ma KW Chen X Li W. MDM2 rs2279744 and TP53 rs1042522 polymorphisms associated with etoposide- and cisplatin-induced grade III/IV neutropenia in Chinese extensive-stage small-cell lung cancer patients. Oncol Res Treat. (2014) 37:176–80. 10.1159/000360785 24732641

[28] Zheng D Chen Y Gao C Wei Y Cao G Lu N. Polymorphisms of p53 and MDM2 genes are associated with severe toxicities in patients with non-small cell lung cancer. Cancer Biol Ther. (2014) 15:1542–51. 10.4161/15384047.2014.956599 25482940 PMC4623062

[29] Gu S Wu Q Zhao X Wu W Gao Z Tan X. Association of CASP3 polymorphism with hematologic toxicity in patients with advanced non-small-cell lung carcinoma treated with platinum-based chemotherapy. Cancer Sci. (2012) 103:1451–9. 10.1111/j.1349-7006.2012.02323.x 22568453 PMC7659371

[30] Ludovini V Floriani I Pistola L Minotti V Meacci M Chiari R. Association of cytidine deaminase and xeroderma pigmentosum group D polymorphisms with response, toxicity, and survival in cisplatin/gemcitabine-treated advanced non-small cell lung cancer patients. J Thorac Oncol. (2011) 6:2018–26. 10.1097/JTO.0b013e3182307e1f 22052224

[31] Wang Z Xu B Lin D Tan W Leaw S Hong X. XRCC1 polymorphisms and severe toxicity in lung cancer patients treated with cisplatin-based chemotherapy in Chinese population. Lung Cancer. (2008) 62:99–104. 10.1016/j.lungcan.2008.02.019 18400332

[32] Booton R Ward T Heighway J Ashcroft L Morris J Thatcher N. Glutathione-S-transferase P1 isoenzyme polymorphisms, platinum-based chemotherapy, and non-small cell lung cancer. J Thorac Oncol. (2006) 1:679–83. 10.1097/01243894-200609000-00013 17409936

[33] Suk R Gurubhagavatula S Park S Zhou W Su L Lynch TJ Polymorphisms in ERCC1 and grade 3 or 4 toxicity in non-small cell lung cancer patients. Clin Cancer Res. (2005) 11:1534–8. 10.1158/1078-0432.CCR-04-1953 15746057

[34] Tibaldi C Giovannetti E Vasile E Mey V Laan AC Nannizzi S. Correlation of CDA, ERCC1, and XPD polymorphisms with response and survival in gemcitabine/cisplatin-treated advanced non-small cell lung cancer patients. Clin Cancer Res. (2008) 14:1797–803. 10.1158/1078-0432.CCR-07-1364 18347182

[35] Peng Y Ma F Tan C Wan X Yi L Peng L. Model-based economic evaluation of ceritinib and platinum-based chemotherapy as first-line treatments for advanced non-small cell lung cancer in China. Adv Ther. (2019) 36:3047–58. 10.1007/s12325-019-01103-4 31576479

[36] Zhang X Zhang D Huang L Li G Chen L Ma J. Discovery of novel biomarkers of therapeutic responses in Han Chinese pemetrexed-based treated advanced NSCLC patients. Front Pharmacol. (2019) 10:944. 10.3389/fphar.2019.00944 31507426 PMC6716463

[37] Weisberg IS Jacques PF Selhub J Bostom AG Chen Z Curtis Ellison R. The 1298A–>C polymorphism in methylenetetrahydrofolate reductase (MTHFR): *in vitro* expression and association with homocysteine. Atherosclerosis. (2001) 156:409–15. 10.1016/s0021-9150(00)00671-7 11395038

[38] Hashemi M Omrani M Eskandari-Nasab E Hasani SS Mashhadi MA Taheri M. A 40-bp insertion/deletion polymorphism of Murine Double Minute2 (MDM2) increased the risk of breast cancer in Zahedan, Southeast Iran. Iran Biomed J. (2014) 18:245–9. 10.6091/ibj.13332.2014 25326024 PMC4225065

[39] Luan L Wang H Zhao B Wang F Shi J Xu X. Association of MDM2 gene SNP 309 polymorphism and human non-small cell lung cancer susceptibility: a meta-analysis. Pathol Res Pract. (2019) 215:152538. 10.1016/j.prp.2019.152538 31326197

[40] Zhuo W Zhang L Zhu B Ling J Chen Z. Association of MDM2 SNP309 variation with lung cancer risk: evidence from 7196 cases and 8456 controls. PLoS ONE. (2012) 7:e41546. 10.1371/journal.pone.0041546 22844496 PMC3402389

[41] Perez-Ramirez C Canadas-Garre M. Pharmacogenetics of platinum-based chemotherapy: impact of DNA repair and folate metabolism gene polymorphisms on prognosis of non-small cell lung cancer patients. Pharmacogenomics J. (2019) 19:164–77. 10.1038/s41397-018-0014-8 29662106

[42] Perez-Ramirez C Canadas-Garre M Molina MA Robles AI Faus-Dader MJ Calleja-Hernandez MA. Contribution of genetic factors to platinum-based chemotherapy sensitivity and prognosis of non-small cell lung cancer. Mutat Res. (2017) 771:32–58. 10.1016/j.mrrev.2016.11.003 28342452

[43] Zhang R Zhou F Cheng L Yu A Zhu M Wang M. Genetic variants in nucleotide excision repair pathway predict survival of esophageal squamous cell cancer patients receiving platinum-based chemotherapy. Mol Carcinog. (2018) 57:1553–65. 10.1002/mc.22877 30035334

[44] Yu J Xiao J Yang Y Cao B. Oxaliplatin-based doublets versus cisplatin or carboplatin-based doublets in the first-line treatment of advanced nonsmall cell lung cancer. Medicine. (2015) 94:e1072. 10.1097/md.0000000000001072 26166081 PMC4504603

[45] Amarasena IU Chatterjee S Walters JA Wood-Baker R Fong KM Platinum versus non-platinum chemotherapy regimens for small cell lung cancer. Cochrane Database Syst Rev. (2015) Cd006849 10.1002/14651858.CD006849.pub3 26233609 PMC7263420

