# Supplementary material for: Genetic Polymorphisms and Platinum-Based Chemotherapy-Induced Toxicities in Patients With Lung Cancer: A Systematic Review and Meta-Analysis
Source: Front Oncol. 2020 Mar 17;9:1573. doi: 10.3389/fonc.2019.01573 (PMC7090160; doi:10.3389/fonc.2019.01573)
Supplement: Supplementary file 1 [file Data_Sheet_1.pdf]

Table S1 Summary of meta-analysis of the association between genetic polymorphisms and platinum induced toxicities in different subgroup.

Gene	SNP	toxicity	Subgroup	No. of studies	Polled OR (95%CI)	P	N	model	I^2%	P-hetero
ERCC1	C118T	Grade 3-4 hematological toxicity	Chinese	2	0.74 [0.36, 1.49]	0.40	802	F	0%	0.74
			Non-Chinese	4	0.83 [0.55, 1.25]	0.36	609	F	0%	0.56
		Grade 3-4 GI toxicity	Total	3	0.80 [0.56, 1.15]	0.23	1450	F	0%	0.81
			Non-Chinese	2	0.81 [0.39, 1.70]	0.58	355	F	0%	0.82
	C8092A	Grade 3-4 hematological toxicity	Chinese	2	0.80 [0.52, 1.21]	0.29	480	F	0%	0.85
			Non-Chinese	2	0.93 [0.63, 1.37]	0.70	557	F	0%	0.54
XRCC1	rs25487	Grade 3-4 hematological toxicity	Total	5	0.94 [0.65, 1.35]	0.72	1366	F	15%	0.32
			Chinese	4	1.08 [0.70, 1.68]	0.73	943	F	8%	0.35
		Grade 3-4 GI toxicity	Total	5	1.29 [0.53, 3.16]	0.57	1366	R	71%	0.009
			Chinese	4	1.02 [0.59, 1.75]	0.96	943	R	71%	0.004
P53	rs1042522	Grade 3-4 hematological toxicity	Total	4	0.82 [0.59, 1.15]	0.25	1033	F	30%	0.23
			Chinese	3	0.77 [0.55, 1.08]	0.13	971	F	0%	0.51
ABCB1	rs1045642	Grade 3-4 hematological toxicity	Chinese	2	1.67 [0.42, 6.60]	0.47	930	R	94%	<0.0001
			Non-Chinese	2	2.32 [1.07, 5.04]	**0.03**	223	F	28%	0.24

			Total	3	1.34 [0.38, 4.75]	0.65	957	R	83%	0.003
		Grade 3-4 GI toxicity	Chinese	2	1.13 [0.25, 5.02]	0.87	803	R	91%	0.0007
ABCB2	rs717620	Grade 3-4 hematological toxicity	Total	3	1.35 [0.43, 4.25]	0.61	923	R	90%	<0.0001
			Chinese	2	1.53 [0.32, 7.28]	0.59	923	R	94%	<0.0001
GSTP1	A313G	Grade 3-4 hematological toxicity	Total	4	1.44 [0.77, 2.7]	0.26	745	F	0%	0.49
			Non-Chinese	3	1.59 [0.28, 2.55]	0.22	312	F	11%	0.33
XPD	rs13181	Grade 3-4 hematological toxicity	Total	4	1.00 [0.55, 1.85]	0.99	742	F	0%	0.75
			Non-Chinese	3	1.00 [0.52, 1.93]	1.00	308	F	0%	0.55
	rs1799793	Grade 3-4 hematological toxicity	Total	3	2.46 [0.46, 13.04]	0.29	548	R	72%	0.03
			Non-Chinese	2	2.79 [0.18, 42.23]	0.46	116	R	86%	0.008
MTHFR	rs1801133	Grade 3-4 GI toxicity	Total	3	1.29 [0.86, 1.92]	0.22	1227	F	0%	0.52
			Chinese	2	1.24 [0.82, 1.86]	0.31	1088	F	0%	0.70
		Grade 3-4 hematological toxicity	Total	3	**1.68 [1.12, 2.52]**	0.01	1229	F	0%	0.40
			Chinese	2	**1.84 [1.15, 2.95]**	**0.01**	1088	F	20%	0.26

Note. F fixed-effects model; R random model; P-hetero P-value for heterogeneity test.

ERCC1 C118T

Study or Subgroup	CC+CT Events	CC+CT Total	TT Events	TT Total	Weight	Odds Ratio M-H, Fixed, 95% CI
**2.1.1 Grade 3-4 Gastrointestinal Toxicity**						
Cristina 2016	2	92	1	49	5.0%	1.07 [0.09, 12.07]
Leiyun Wang 2018	65	407	6	28	37.2%	0.70 [0.27, 1.79]
Rebecca Suk 2005	11	79	23	135	57.7%	0.79 [0.36, 1.72]
**Subtotal (95% CI)**		**578**		**212**	**100.0%**	**0.77 [0.43, 1.38]**
Total events	78		30			
Heterogeneity: Chi² = 0.12, df = 2 (P = 0.94); I² = 0%						
Test for overall effect: Z = 0.88 (P = 0.38)						
**2.1.2 Grade 3-4 Hematologic toxicity**						
CarmeloTibaldi 2008	3	47	0	18	1.3%	2.91 [0.14, 59.18]
Cristina 2016	22	92	12	49	23.8%	0.97 [0.43, 2.17]
Leiyun Wang 2018	98	407	8	28	0.0%	0.79 [0.34, 1.86]
Rebecca Suk 2005	31	79	58	135	52.0%	0.86 [0.49, 1.51]
Vienna Ludovini 2011	23	158	8	31	22.8%	0.49 [0.20, 1.23]
Yi Zheng 2017	28	380	3	26	0.0%	0.61 [0.17, 2.16]
**Subtotal (95% CI)**		**376**		**233**	**100.0%**	**0.83 [0.55, 1.25]**
Total events	79		78			
Heterogeneity: Chi² = 2.08, df = 3 (P = 0.56); I² = 0%						
Test for overall effect: Z = 0.91 (P = 0.36)						

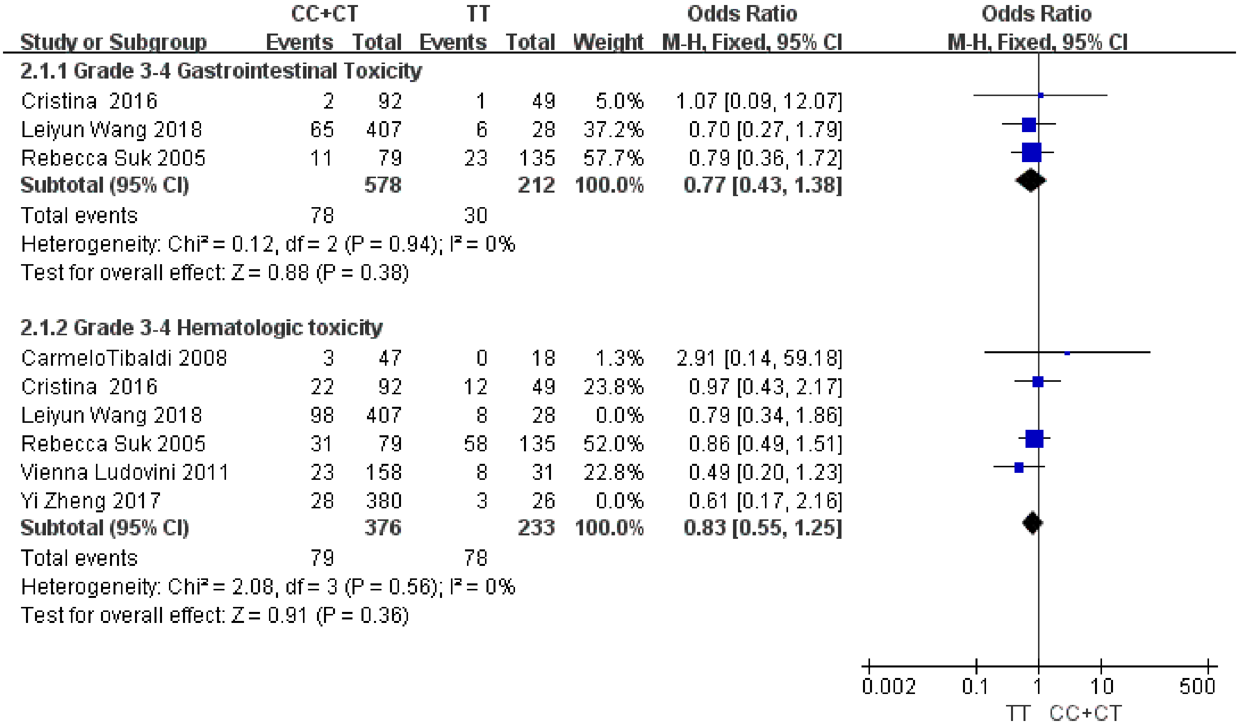

ERCC1 C8092A

Study or Subgroup	GG Events	GG Total	GA+TT Events	GA+TT Total	Weight	Odds Ratio M-H, Fixed, 95% CI
**2.2.1 Grade 3-4 hematology toxicity**						
Cristina 2016	45	184	57	232	72.0%	0.99 [0.63, 1.56]
Leiyun Wang 2018	42	185	64	240	0.0%	0.81 [0.52, 1.26]
T. Powro´zek 2016	16	74	18	67	28.0%	0.75 [0.35, 1.63]
Yi Zheng 2017	5	23	9	32	0.0%	0.71 [0.20, 2.49]
**Subtotal (95% CI)**		**258**		**299**	**100.0%**	**0.93 [0.63, 1.37]**
Total events	61		75			
Heterogeneity: Chi² = 0.38, df = 1 (P = 0.54); I² = 0%						
Test for overall effect: Z = 0.39 (P = 0.70)						
**2.2.2 Grade 3-4 nephrotoxicity**						
Cristina 2016	2	74	1	67	12.4%	1.83 [0.16, 20.69]
T. Powro´zek 2016	5	23	11	32	87.6%	0.53 [0.15, 1.81]
**Subtotal (95% CI)**		**97**		**99**	**100.0%**	**0.69 [0.24, 2.01]**
Total events	7		12			
Heterogeneity: Chi² = 0.80, df = 1 (P = 0.37); I² = 0%						
Test for overall effect: Z = 0.68 (P = 0.50)						

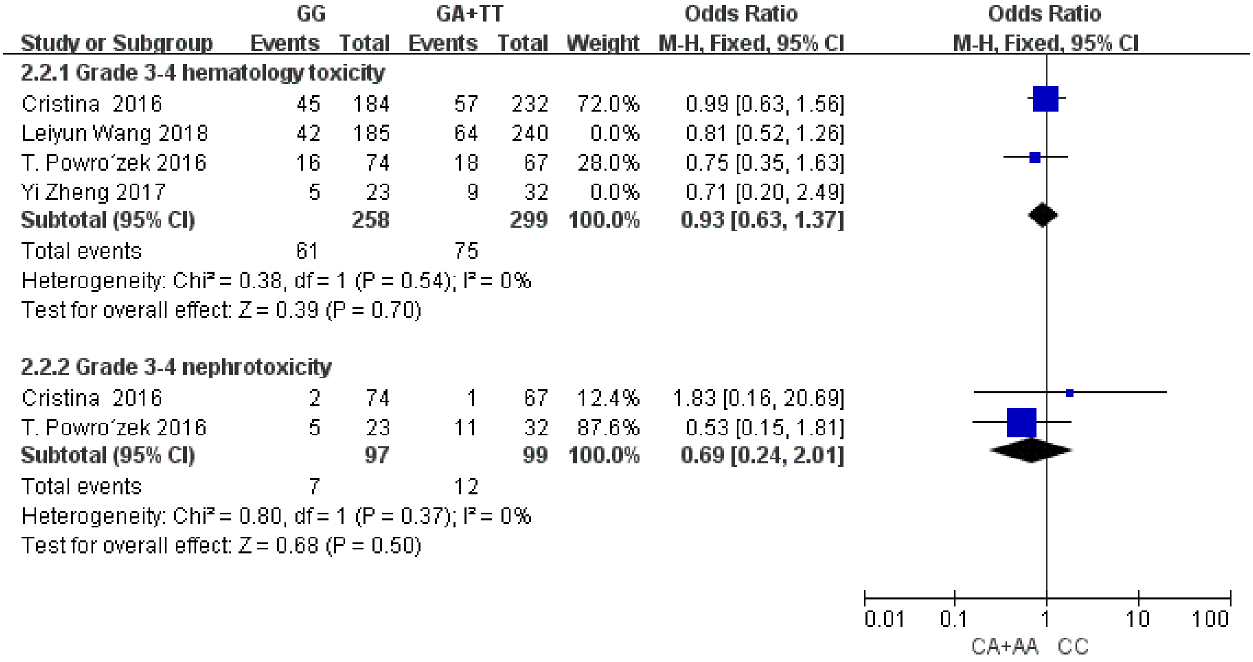

Figure S1. Forest plot of the meta-analysis for association between *ERCC1* C118T, C8092A polymorphisms and platinum-based chemotherapy toxicities.

Study or Subgroup	AA Events	AA Total	AG+GG Events	AG+GG Total	Weight	Odds Ratio M-H, Fixed, 95% CI
**5.1.1 Grade 3-4 gastrointestinal toxicity**						
Cristina 2016	18	211	20	212	41.6%	0.90 [0.46, 1.75]
Leiyun Wang 2018	5	31	65	397	18.0%	0.98 [0.36, 2.65]
Yi Zheng 2017	1	20	2	121	1.2%	3.13 [0.27, 36.25]
Yu Peng 2014	6	9	41	226	2.4%	9.02 [2.17, 37.58]
Zhonghua Wang 2008	11	77	17	62	36.8%	0.44 [0.19, 1.03]
**Subtotal (95% CI)**		**348**		**1018**	**100.0%**	**0.97 [0.63, 1.47]**
Total events	41		145			
Heterogeneity: Chi² = 13.64, df = 4 (P = 0.009); I² = 71%						
Test for overall effect: Z = 0.16 (P = 0.87)						
**5.1.2 Grade 3-4 hematological toxicity**						
Cristina 2016	17	211	24	212	36.6%	0.69 [0.36, 1.32]
Leiyun Wang 2018	10	31	95	397	15.5%	1.51 [0.69, 3.33]
Yi Zheng 2017	4	20	40	121	15.1%	0.51 [0.16, 1.61]
Yu Peng 2014	5	9	86	226	4.9%	2.03 [0.53, 7.79]
Zhonghua Wang 2008	32	77	26	62	28.0%	0.98 [0.50, 1.94]
**Subtotal (95% CI)**		**348**		**1018**	**100.0%**	**0.94 [0.65, 1.35]**
Total events	68		271			
Heterogeneity: Chi² = 4.69, df = 4 (P = 0.32); I² = 15%						
Test for overall effect: Z = 0.35 (P = 0.72)						

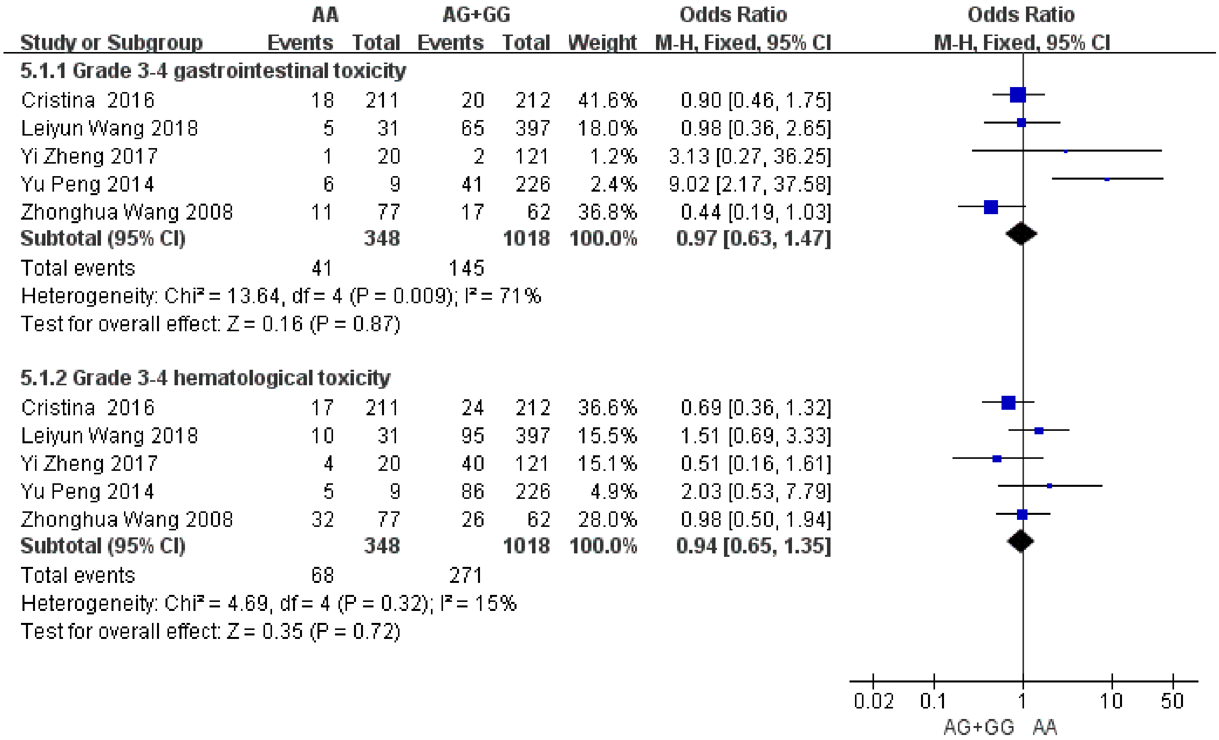

Figure S2. Forest plot of the meta-analysis for association between *XRCC1* G1196A polymorphism and platinum-based chemotherapy toxicities.

Study or Subgroup	GG+GC Events	GG+GC Total	CC Events	CC Total	Weight	Odds Ratio M-H, Fixed, 95% CI
**8.1.1 P53 Arg72Pro and grade 3-4 hematologic toxicity**						
Datong Zheng 2014	28	90	8	29	10.8%	1.19 [0.47, 3.00]
Leiyun Wang 2018	79	331	22	80	34.8%	0.83 [0.48, 1.44]
Vienna Ludovini 2011	31	58	0	4	0.6%	10.31 [0.53, 200.18]
Xu Wang 2014	172	356	50	85	53.9%	0.65 [0.41, 1.06]
**Subtotal (95% CI)**		**835**		**198**	**100.0%**	**0.82 [0.59, 1.15]**
Total events	310		80			
Heterogeneity: Chi² = 4.27, df = 3 (P = 0.23); I² = 30%						
Test for overall effect: Z = 1.15 (P = 0.25)						

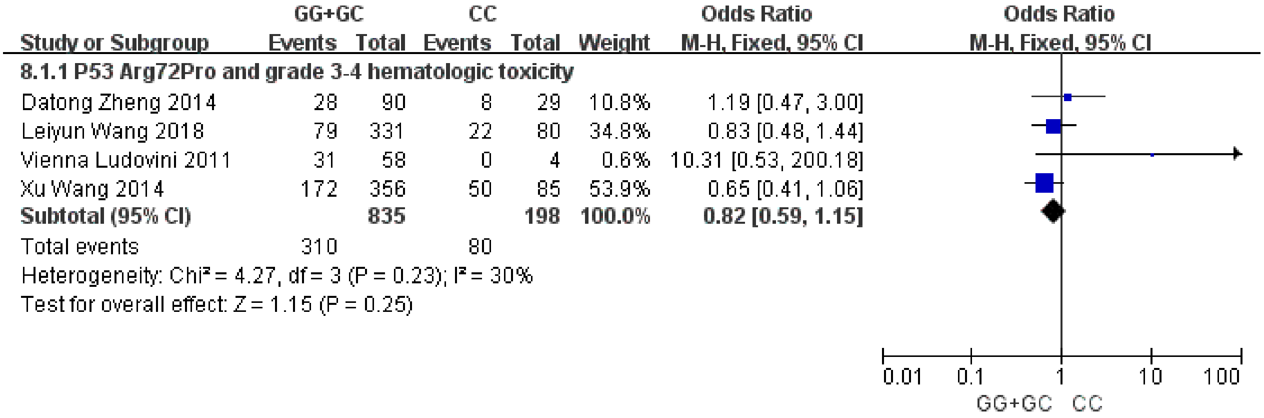

Figure S3. Forest plot of the meta-analysis for association between *P53* Arg72Pro polymorphism and platinum-based chemotherapy toxicities.

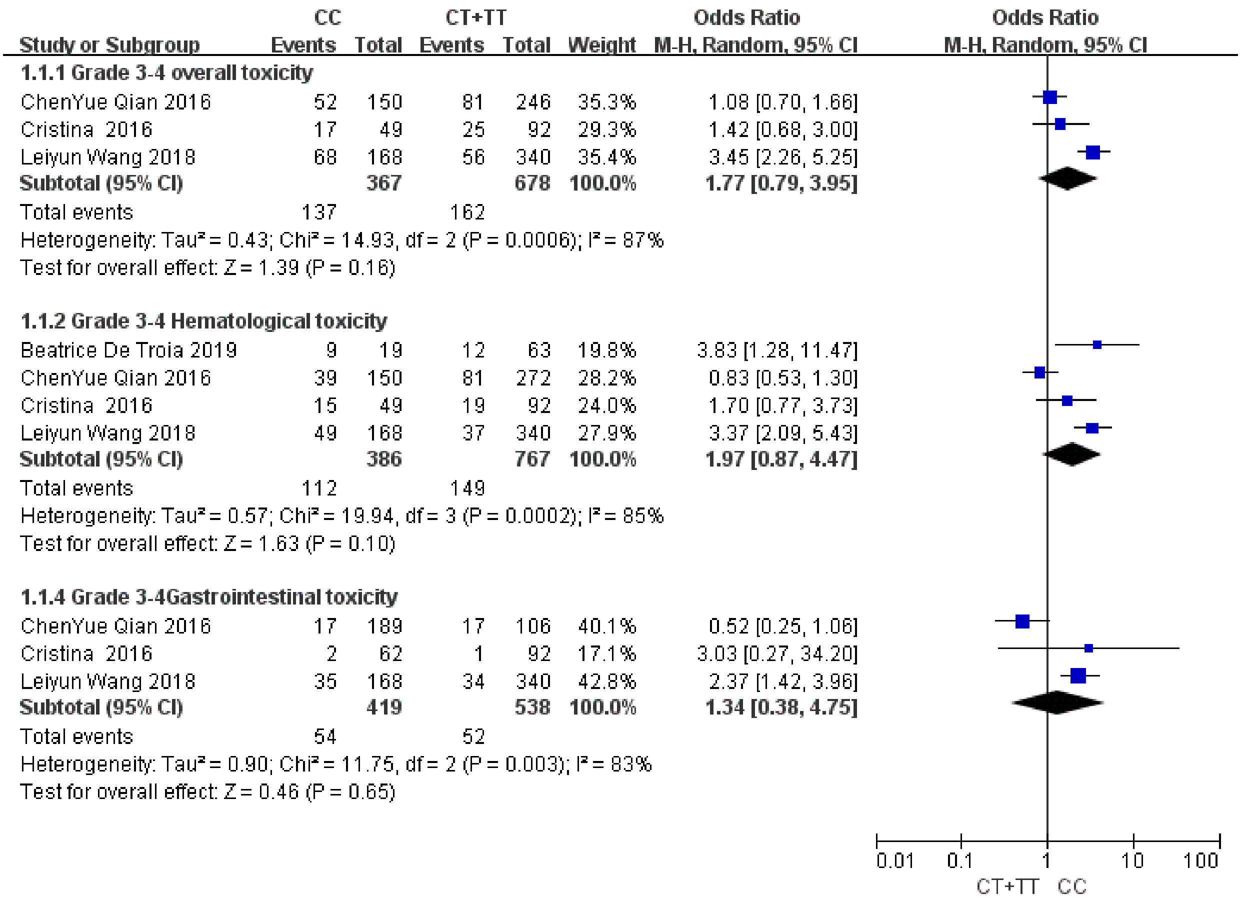

Figure S4. Forest plot of the meta-analysis for association between *ABCB1* G2677T/A polymorphism and platinum-based chemotherapy toxicities.

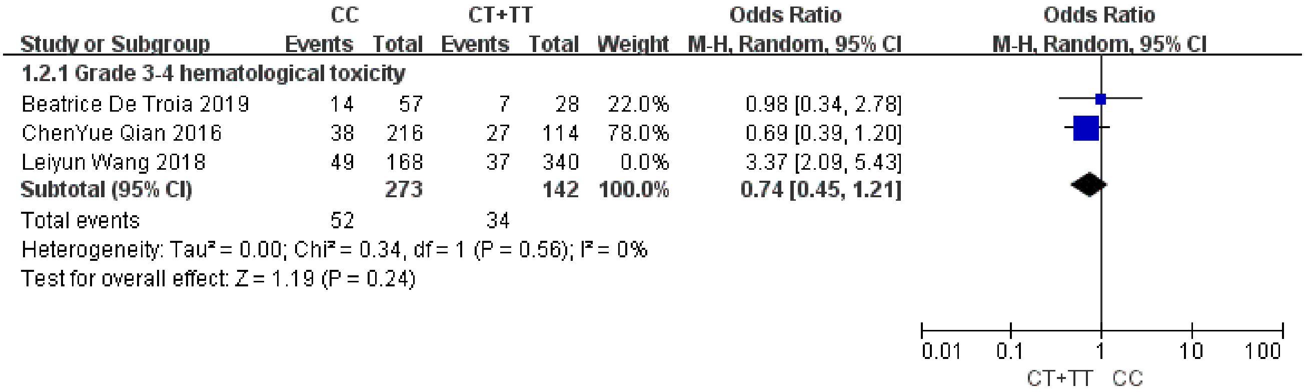

Figure S5. Forest plot of the meta-analysis for association between *ABCB2* -24C>T polymorphism and platinum-based chemotherapy toxicities.

Study or Subgroup	AA+AG Events	AA+AG Total	GG Events	GG Total	Weight	Odds Ratio M-H, Fixed, 95% CI
Beatrice De Troia 2019	20	72	1	10	7.4%	3.46 [0.41, 29.11]
Leiyun Wang 2018	102	415	4	18	33.9%	1.14 [0.37, 3.54]
Markus Joerger 2011	61	78	9	15	19.3%	2.39 [0.75, 7.67]
Richard Booten 2006	23	115	5	22	39.4%	0.85 [0.28, 2.55]
**Total (95% CI)**		**680**		**65**	**100.0%**	**1.44 [0.77, 2.70]**
Total events	206		19			

Heterogeneity: Chi² = 2.43, df = 3 (P = 0.49); I² = 0%
Test for overall effect: Z = 1.14 (P = 0.26)

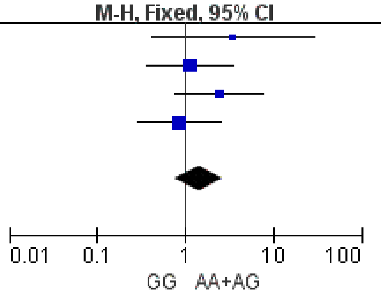

Figure S6. Forest plot of the meta-analysis for association between *GSTP1* A313G polymorphism and platinum-based chemotherapy toxicities.

XPD A2251C

Study or Subgroup	AA Events	AA Total	AC+CC Events	AC+CC Total	Weight	Odds Ratio M-H, Fixed, 95% CI
**4.1.1 Grade 3-4 haematological toxicity**						
CarmeloTibaldi 2008	2	20	1	34	3.2%	3.67 [0.31, 43.27]
Leiyun Wang 2018	2	8	104	426	13.9%	1.03 [0.21, 5.19]
T. Powro´zek 2016	9	57	22	132	53.9%	0.94 [0.40, 2.19]
Vienna Ludovini 2011	5	20	13	45	29.0%	0.82 [0.25, 2.72]
**Subtotal (95% CI)**		**105**		**637**	**100.0%**	**1.00 [0.55, 1.85]**
Total events	18		140			

Heterogeneity: Chi² = 1.19, df = 3 (P = 0.75); I² = 0%
Test for overall effect: Z = 0.01 (P = 0.99)

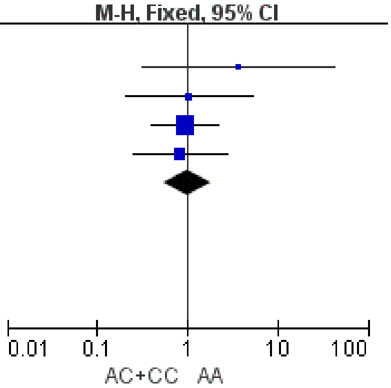

XPD G934A

Study or Subgroup	AA Events	AA Total	AG+GG Events	AG+GG Total	Weight	Odds Ratio M-H, Random, 95% CI
**4.2.1 Grade 3-4 haematological toxicity**						
CarmeloTibaldi 2008	5	9	4	42	31.9%	11.88 [2.23, 63.10]
Leiyun Wang 2018	2	5	104	427	30.3%	2.07 [0.34, 12.56]
T. Powro´zek 2016	5	21	13	44	37.8%	0.75 [0.23, 2.46]
**Subtotal (95% CI)**		**35**		**513**	**100.0%**	**2.46 [0.46, 13.04]**
Total events	12		121			

Heterogeneity: Tau² = 1.55; Chi² = 7.02, df = 2 (P = 0.03); I² = 72%
Test for overall effect: Z = 1.06 (P = 0.29)

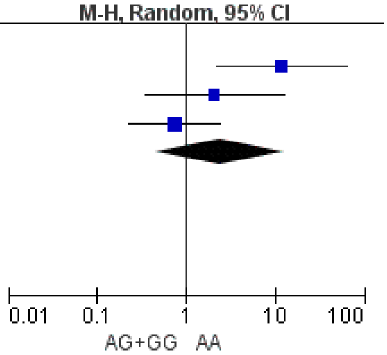

Figure S7. Forest plot of the meta-analysis for association between *XPD* A2251C and G934A polymorphisms and platinum-based chemotherapy toxicities.

***MTHFR* C677T**

Study or Subgroup	CC Events	CC Total	CT+TT Events	CT+TT Total	Weight	Odds Ratio M-H, Fixed, 95% CI
**7.1.1 Grade 3-4 gastrointestinal toxicity**						
Cristina 2016	2	42	1	97	1.4%	4.80 [0.42, 54.45]
Leiyun Wang 2018	13	88	16	116	28.6%	1.08 [0.49, 2.39]
Xiaoying Li 2014	30	284	50	600	70.0%	1.30 [0.81, 2.09]
**Subtotal (95% CI)**		**414**		**813**	**100.0%**	**1.29 [0.86, 1.92]**
Total events	45		67			
Heterogeneity: Chi² = 1.31, df = 2 (P = 0.52); I² = 0%						
Test for overall effect: Z = 1.23 (P = 0.22)						
**7.1.2 Grade 3-4 hematological toxicity**						
Cristina 2016	12	44	22	97	28.8%	1.28 [0.57, 2.89]
Leiyun Wang 2018	24	88	24	116	43.4%	1.44 [0.75, 2.75]
Xiaoying Li 2014	18	284	16	600	27.8%	2.47 [1.24, 4.92]
**Subtotal (95% CI)**		**416**		**813**	**100.0%**	**1.68 [1.12, 2.52]**
Total events	54		62			
Heterogeneity: Chi² = 1.85, df = 2 (P = 0.40); I² = 0%						
Test for overall effect: Z = 2.49 (P = 0.01)						

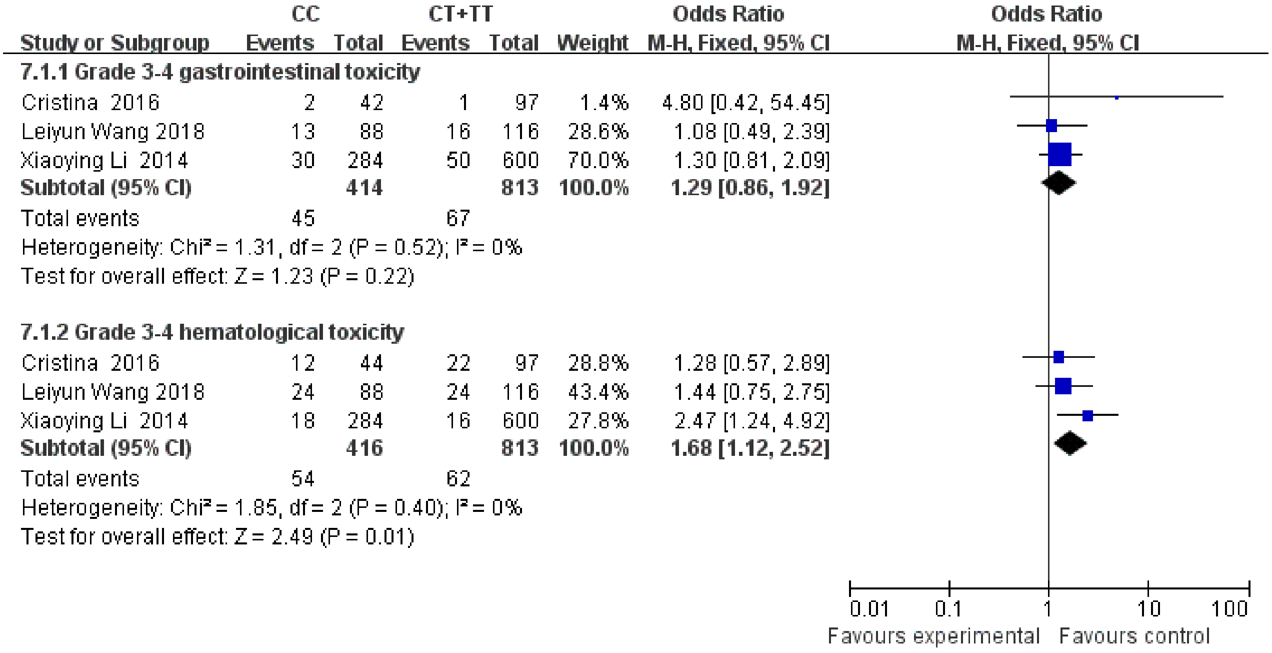

***MTHFR* C677T**

Study or Subgroup	AA Events	AA Total	AC+CC Events	AC+CC Total	Weight	Odds Ratio M-H, Fixed, 95% CI
**7.2.1 Grade 3-4 gastrointestinal toxicity**						
Cristina 2016	1	75	2	66	9.2%	0.43 [0.04, 4.88]
Xiaoying Li 2014	63	651	17	312	90.8%	1.86 [1.07, 3.23]
**Subtotal (95% CI)**		**726**		**378**	**100.0%**	**1.73 [1.02, 2.94]**
Total events	64		19			
Heterogeneity: Chi² = 1.32, df = 1 (P = 0.25); I² = 24%						
Test for overall effect: Z = 2.02 (P = 0.04)						
**7.2.2 Grade 3-4 hematological toxicity**						
Cristina 2016	16	75	18	66	46.8%	0.72 [0.33, 1.57]
Xiaoying Li 2014	21	645	13	304	53.2%	0.75 [0.37, 1.53]
**Subtotal (95% CI)**		**720**		**370**	**100.0%**	**0.74 [0.44, 1.24]**
Total events	37		31			
Heterogeneity: Chi² = 0.01, df = 1 (P = 0.94); I² = 0%						
Test for overall effect: Z = 1.14 (P = 0.26)						

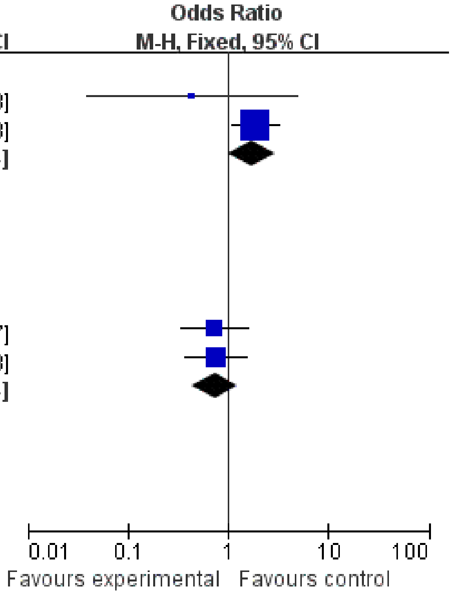

Figure S8. Forest plot of the meta-analysis for association between *MTHFR* A1298C, C677T polymorphisms and platinum-based chemotherapy toxicities.

MDM2 rs1470383

Study or Subgroup	GG Events	GG Total	GT+TT Events	GT+TT Total	Weight	Odds Ratio M-H, Random, 95% CI
**6.1.1 Grade 3-4 hematologic toxicity**						
Datong Zheng 2014	8	61	52	160	24.9%	0.31 [0.14, 0.71]
Leiyun Wang 2018	23	102	80	317	28.6%	0.86 [0.51, 1.46]
Xu Wang 2014	5	33	31	86	21.7%	0.32 [0.11, 0.90]
Yi Zheng 2017	11	109	15	321	24.9%	2.29 [1.02, 5.15]
**Subtotal (95% CI)**		**305**		**884**	**100.0%**	**0.69 [0.29, 1.62]**
Total events	47		178			
Heterogeneity: Tau² = 0.60; Chi² = 14.70, df = 3 (P = 0.002); I² = 80%						
Test for overall effect: Z = 0.85 (P = 0.39)						

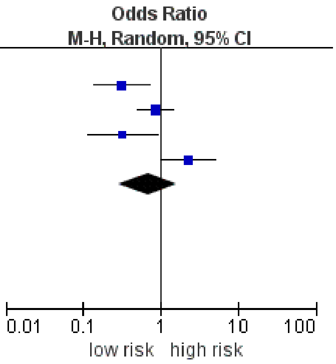

MDM2 rs2279744

Study or Subgroup	AA Events	AA Total	AG+GG Events	AG+GG Total	Weight	Odds Ratio M-H, Fixed, 95% CI
**6.2.1 Grade 3-4 overall toxicity**						
Cristina 2016	31	99	11	42	14.9%	1.28 [0.57, 2.88]
Ji Qian 2015	137	448	63	197	85.1%	0.94 [0.65, 1.34]
**Subtotal (95% CI)**		**547**		**239**	**100.0%**	**0.99 [0.71, 1.37]**
Total events	168		74			
Heterogeneity: Chi² = 0.49, df = 1 (P = 0.48); I² = 0%						
Test for overall effect: Z = 0.07 (P = 0.95)						
**6.2.2 Grade 3-4 Hematologic toxicity**						
Cristina 2016	26	99	8	42	12.4%	1.51 [0.62, 3.69]
Ji Qian 2015	110	448	56	197	87.6%	0.82 [0.56, 1.19]
**Subtotal (95% CI)**		**547**		**239**	**100.0%**	**0.91 [0.64, 1.28]**
Total events	136		64			
Heterogeneity: Chi² = 1.55, df = 1 (P = 0.21); I² = 35%						
Test for overall effect: Z = 0.56 (P = 0.57)						
**6.2.3 Grade 3-4 gastrointestinal toxicity**						
Cristina 2016	3	99	0	42	3.5%	3.08 [0.16, 61.00]
Ji Qian 2015	43	448	15	197	96.5%	1.29 [0.70, 2.38]
**Subtotal (95% CI)**		**547**		**239**	**100.0%**	**1.35 [0.74, 2.46]**
Total events	46		15			
Heterogeneity: Chi² = 0.32, df = 1 (P = 0.57); I² = 0%						
Test for overall effect: Z = 0.98 (P = 0.33)						

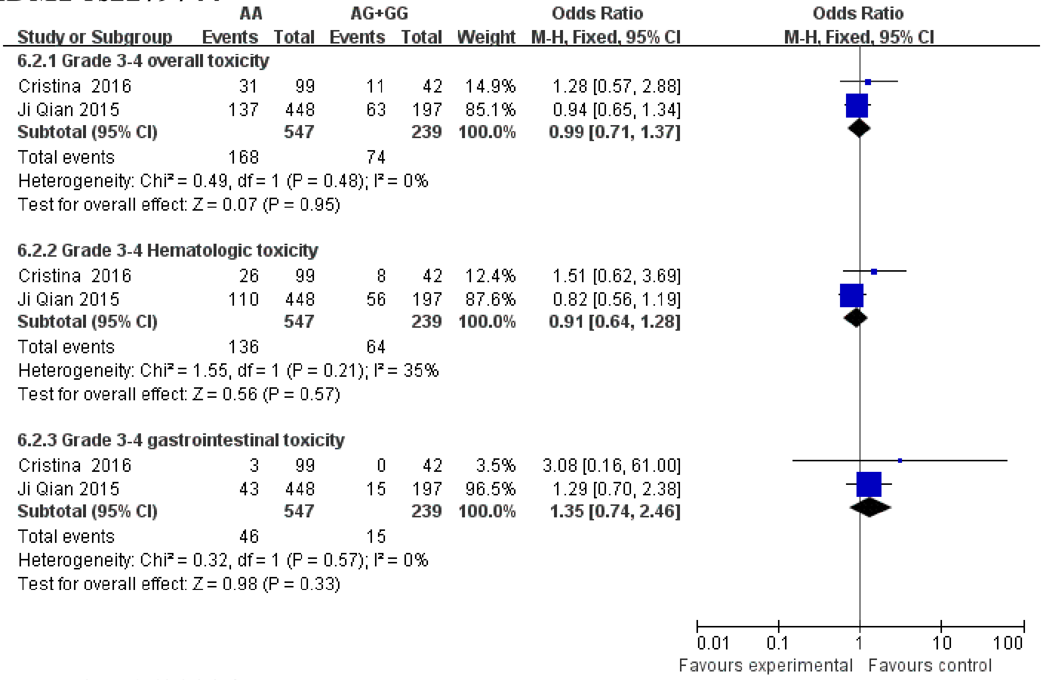

MDM2 rs1690924

Study or Subgroup	TT Events	TT Total	TC+CC Events	TC+CC Total	Weight	Odds Ratio M-H, Fixed, 95% CI
**6.3.1 Grade 3-4 overall toxicity**						
Cristina 2016	7	24	35	117	9.9%	0.96 [0.37, 2.53]
Ji Qian 2015	96	333	104	312	90.1%	0.81 [0.58, 1.13]
**Subtotal (95% CI)**		**357**		**429**	**100.0%**	**0.83 [0.60, 1.13]**
Total events	103		139			
Heterogeneity: Chi² = 0.11, df = 1 (P = 0.74); I² = 0%						
Test for overall effect: Z = 1.19 (P = 0.23)						
**6.3.2 Grade 3-4 Hematologic toxicity**						
Cristina 2016	4	19	30	91	12.0%	0.54 [0.17, 1.78]
Ji Qian 2015	87	333	79	312	88.0%	1.04 [0.73, 1.49]
**Subtotal (95% CI)**		**352**		**403**	**100.0%**	**0.98 [0.70, 1.38]**
Total events	91		109			
Heterogeneity: Chi² = 1.07, df = 1 (P = 0.30); I² = 7%						
Test for overall effect: Z = 0.10 (P = 0.92)						
**6.3.3 Grade 3-4 gastrointestinal toxicity**						
Cristina 2016	0	19	3	118	2.7%	0.85 [0.04, 17.03]
Ji Qian 2015	21	333	37	312	97.3%	0.50 [0.29, 0.88]
**Subtotal (95% CI)**		**352**		**430**	**100.0%**	**0.51 [0.29, 0.88]**
Total events	21		40			
Heterogeneity: Chi² = 0.11, df = 1 (P = 0.74); I² = 0%						
Test for overall effect: Z = 2.40 (P = 0.02)						

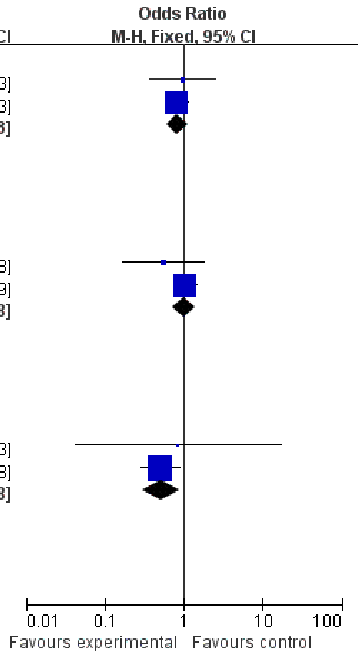

Figure S9. Forest plot of the meta-analysis for association between *MDM2* polymorphisms and platinum-based chemotherapy toxicities.

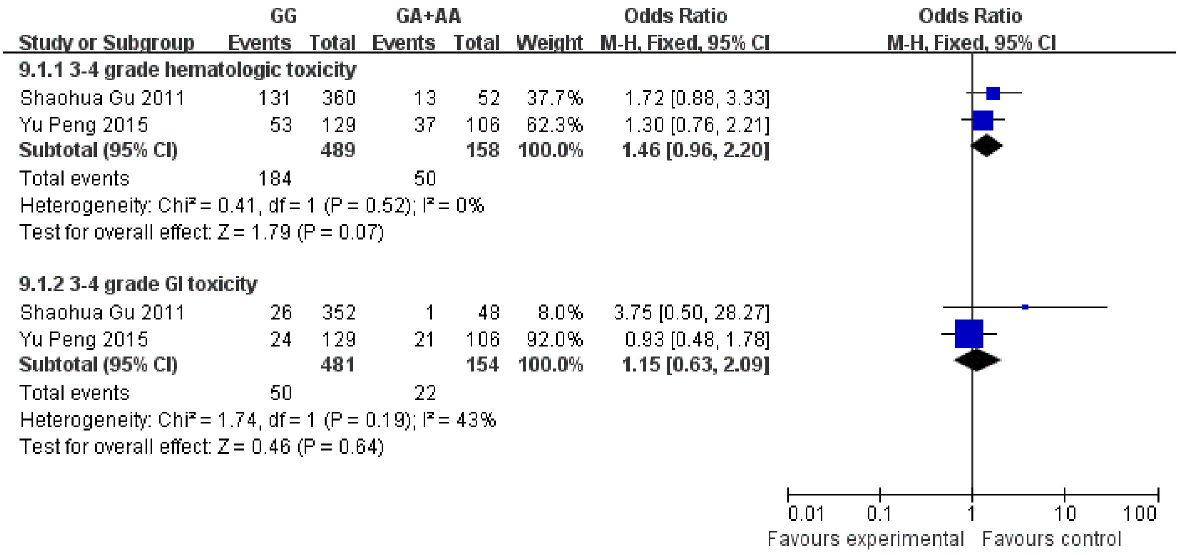

Figure S10. Forest plot of the meta-analysis for association between *BAX rs4645878* mutation and platinum-based chemotherapy toxicities.

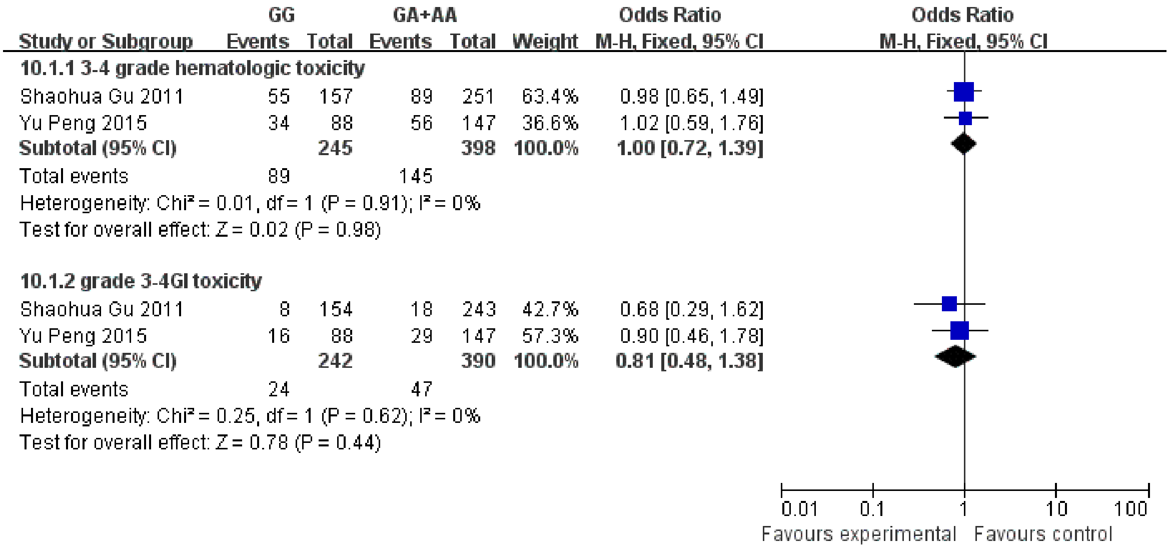

Figure S11. Forest plot of the meta-analysis for association between *BCL2 rs2279115* mutation and platinum-based chemotherapy toxicities.
